# Changes in the Diaphragm Lipid Content after Administration of Streptozotocin and High-Fat Diet Regime

**DOI:** 10.1155/2017/3437169

**Published:** 2017-11-06

**Authors:** Bartlomiej Lukaszuk, Agnieszka Miklosz, Malgorzata Zendzian-Piotrowska, Beata Wojcik, Jan Gorski, Adrian Chabowski

**Affiliations:** ^1^Department of Physiology, Medical University of Bialystok, Bialystok, Poland; ^2^Department of Hygiene, Epidemiology and Ergonomics, Medical University of Bialystok, Bialystok, Poland

## Abstract

The diaphragm is a dome-shaped skeletal muscle indispensable for breathing. Its activity contributes up to 70% of the total ventilatory function at rest. In comparison to other skeletal muscles, it is distinguished by an oxidative phenotype and uninterrupted cyclic contraction pattern. Surprisingly, the research regarding diaphragm diabetic phenotype particularly in the light of lipid-induced insulin resistance is virtually nonexistent. Male Wistar rats were randomly allocated into 3 groups: control, streptozotocin-induced (STZ) type-1 diabetes, and rodents fed with high-fat diet (HFD). Additionally, half of the animals from each group were administered with myriocin, a robust, selective inhibitor of ceramide synthesis and, therefore, a potent agent ameliorating insulin resistance. Diaphragm lipid contents were evaluated using chromatography. Fatty acid transporter expression was determined by Western blot. The STZ and HFD rats had increased concentration of lipids, namely, ceramides (CER) and diacylglycerols (DAG). Interestingly, this coincided with an increased concentration of long-chain (C ≥ 16) saturated fatty acid species present in both the aforementioned lipid fractions. The CER/DAG accumulation was accompanied by an elevated fatty acid transporter expression (FATP-1 in HFD and FATP-4 in STZ). Surprisingly, we observed a significantly decreased triacylglycerol content in the diaphragms of STZ-treated rats.

## 1. Introduction

Diabetes is a predominant, usually life-long health condition commonly identified by an increased blood glucose level (hyperglycemia) arising in the wake of defects in insulin action and/or secretion [1]. As for the year 2014, it affected over 420 million of adult individuals worldwide, that is approximately 1 in every 11 adult people, and the number is expected to grow in the foreseeable future [2, 3]. Unfortunately, untreated diabetes may well lead to kidney failure, heart attack, stroke, blindness, or even foot amputation [4]. Therefore, unsurprisingly, much research has been conducted in order to better understand the origin of the disease and to devise potential treatments. In the light of the above, special interest has been placed on skeletal muscles, that is, a major contributor (even up to 80%) to postprandial glucose uptake [5, 6]. Interestingly, many scientific reports indicate a disparity between an inordinate myocyte free fatty acid (FFA) inflow and their deficient oxidation rate [5, 6]. As a result, intramyocellular lipid (IMCL) accumulation, mainly in the triacylglycerol (TAG), diacylglycerol (DAG), and ceramide (CER) fraction, takes place [5, 7–9]. Moreover, some of the lipids, that is, DAG and CER, may directly interfere with myocellular insulin signal transduction [5, 9–11]. The phenomenon of insulin resistance is a key pathogenic factor of type 2 diabetes (T2DM). Interestingly, similar changes with respect to muscular lipid content were demonstrated also for insulin-dependent diabetes [12–15]. Perseghin et al., for instance, confirmed the reduction of the insulin-stimulated glucose clearance in the muscles of patients with type 1 diabetes mellitus (T1DM) and its connection with their increased IMCL content [14]. Moreover, the authors found a positive correlation (*r* ≈ 0.75, *r*^2^ = 0.57, *p* < 0.001) between soleus IMCL level and blood glycated hemoglobin HbA1C (commonly used as an indicator of glycemia level in diabetes) [14, 16]. Nevertheless, in the case of insulin-dependent diabetes, lipid accumulation in skeletal muscles is still considered as a secondary alteration [14, 17].

The diaphragm is a dome-shaped sheet of skeletal muscle indispensable for breathing process. Its constantly repeated activity contributes up to 70% of the total ventilatory function at rest [18]. In comparison with other skeletal muscles, the diaphragm is distinguished by a highly oxidative phenotype [19–21]. In Wistar rats, it is composed from the red (≈40%), intermediate (≈35%), and white muscle fibers (≈25%) [20]. Furthermore, the diaphragm is characterized by an uninterrupted cyclic contraction pattern [18]. Therefore, the results obtained for the diaphragm are often compared to those acquired from oxidative and/or oxidative-glycolytic skeletal muscles (i.e., soleus or red gastrocnemius) [22, 23] and, less often, the heart (due to its rhythmicity and oxidative potential) [24, 25]. Surprisingly, the research regarding diaphragm diabetic phenotype particularly in the light of lipid-induced insulin resistance (IR) is virtually nonexistent, with previous studies focused rather on an examination of the muscle strength in diabetes [18, 26].

Therefore, the aim of this study was to investigate the diaphragm lipid content and FA composition in relation to altered insulin levels. For this reason, we adopted streptozotocin injection (STZ) and high-fat diet feeding (HFD), two commonly accepted models of type 1 diabetes and preceding type 2 diabetes insulin resistance, respectively [27, 28]. In our research, we evaluated diaphragm TAG, DAG, and CER (i.e., well-known markers and/or inducers of skeletal muscle tissue IR) total content, as well as their FA composition [9, 10]. Moreover, the above was complemented by an analysis of the diaphragm expression of fatty acid transporters (FAT/CD36, FATP-1, and FATP-4). Additionally, we decided on application of myriocin, that is, an inhibitor of ceramide de novo synthesis and therefore a potent agent for the amelioration of IR [12, 29, 30]. This opened a possibility to determine the potential for a reversal of the induced changes.

## 2. Materials and Methods

### 2.1. Animals and Study Design

All experiments were performed in accordance with the guidelines of the Ethical Committee for Animal Experiments at the Medical University of Bialystok. Male Wistar rats (*n* = 6 per group) were kept under controlled conditions (21°C ± 2, 12 h light/12 h dark cycle) with unlimited access to water and to a commercial chow. The rats were randomly divided into six groups:

1. Control (Ctrl M−)
2. Myriocin (Ctrl M+)
3. Streptozotocin-induced type 1 diabetes (STZ M−)
4. Streptozotocin-induced type 1 diabetes + myriocin (STZ M+)
5. HFD-induced type 2 diabetes (HFD M−)
6. HFD-induced type 2 diabetes + myriocin (HFD M+)

The control group (Ctrl M−) was composed of animals maintained on a standard rodent diet (10% kcal from fat, Research Diets, number D12450B). The animals assigned to the “Ctrl M+” group were treated with myriocin (Sigma-Aldrich, St. Louis, MO), which was injected intraperitoneally at a dose of 0.3 mg/kg of body weight, daily for 7 days. The rats assigned to the “STZ M−” and “STZ M+” groups were given streptozotocin (single dose). The drug (Sigma-Aldrich, St. Louis, MO) was dissolved in citric buffer (pH 4.5) and administered at a dose of 80 mg/kg of body weight. Additionally, the rats in the “STZ M+” group were treated with myriocin (for 7 days after the first week since diabetes onset). The animals allocated to the “HFD M−” group were on a high-fat diet (60% kcal from fat, Research Diets Inc., number D12492) for 5 weeks. Similarly, the rodents allocated to the “HFD M+” group received high-fat diet for 5 weeks and additionally were injected with myriocin for 7 days (since the fourth week of maintaining on a diet). The animals underwent overnight fasting before sacrifice and tissue collection procedures. The rats were anesthetized by intraperitoneal application of pentobarbital with a dose of 80 mg/kg of body weight. Then the samples of the diaphragm were excised, immediately freeze-clamped with aluminum tongs precooled in liquid nitrogen. Blood taken from the abdominal aorta was centrifuged in order to obtain plasma. In between procedures, all the samples were stored at −80°C.

### 2.2. Plasma Insulin and Glucose Level

Insulin level was measured in plasma, with commercially available ELISA kit, according to the manufacturer's instruction (Abbot, USA). Fasting blood glucose concentration was measured with Accu-Chek (Bayer, Germany) glucose meter.

### 2.3. Plasma and Diaphragm Lipid Concentrations

**Figure 1:**
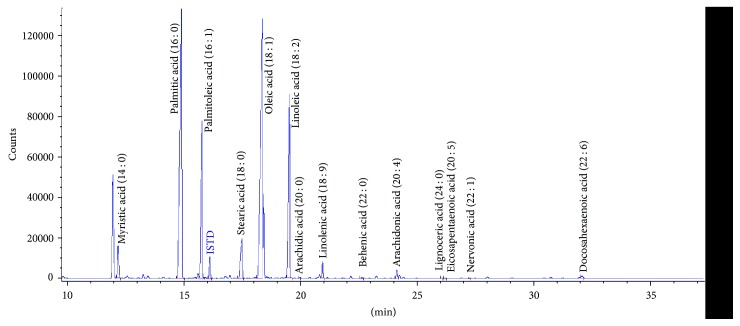
An exemplary chromatogram of fatty acids composing triacylglycerol in the diaphragm of a control rat. The peak to the left of myristic acid (14 : 0) is an antioxidant and was not analyzed. ISTD: internal standard, that is, triheptadecanoin (C 17 : 0).

Lipid content (CER, FFA, DAG, and TAG) was analyzed by gas-liquid chromatography as described previously [31–33]. The studied lipid fractions were extracted using Bligh and Dyer's method [34]. The tissue, powdered under liquid nitrogen, and plasma (200 *μ*l) samples were transferred into glass tubes containing 2 ml of methanol with antioxidant (0.01% butylated hydroxytoluene) and 4 ml of chloroform. Additionally, an internal standard (100 *μ*l) containing heptadecanoic acid (C17:0 FFA), 1,2-diheptadecanoin (C17:0 DAG), and triheptadecanoin (C17:0 TAG) was added. After 24 h, 1.5 ml of water was added to separate lipid layer. Lipids dissolved in chloroform were evaporated under nitrogen stream (37°C) and redissolved in 100 *μ*l of chloroform-methanol solution (2/1, vol/vol). Then the samples were separated using thin-layer chromatography (TLC). Briefly, lipids were fractioned on silica gel plates (silica plate 60, 0.25 mm; Merck) with a diethyl ether : hexane : acetic acid (90 : 10 : 1, vol/vol/vol) resolving solution for CER, DAG, and TAG, while FFA separation was performed in a solvent containing heptane, isopropyl, and acetic acid (60 : 40 : 3, vol/vol/vol) [35]. For the visualization purpose, dried silica plates were sprayed using 2′7′-dichlorofluorscein (0.2% solution) in methanol and exposed to ammonia vapors, and bands were visualized under ultraviolet light. Ceramide class of the lipids was scraped off the plates (according to an appropriate standard, Sigma-Aldrich, St. Louis, MO) and transferred into screw tubes containing pentadecanoic acid (C15:0, Sigma-Aldrich, St. Louis, MO) as an internal standard and transmethylated (14% boron trifluoride-methanol solution). Also, FFAs were transmethylated with BF3/methanol [36]. DAGs were three times eluted using chloroform-methanol solution (9/1, vol/vol), and then an organic phase was evaporated under nitrogen stream and redissolved in BF3/methanol solution [37]. The elution of TAG was done using hexane-diethyl ether solution (1/1, vol/vol) followed by the addition of 2 ml of water. Next, an organic phase was transferred into new glass tubes and evaporated. TAGs were redissolved in diethyl ether and methyl acetate, and sodium methoxide (1 M) and solution of oxalic acid in diethyl ether were added [37]. The fatty acid methyl esters (FAMEs) were extracted using pentane. Then samples were dissolved in hexane and analyzed using a Hewlett-Packard 5890 series II gas chromatograph, an Agilent J&W CP-Sil 88 capillary column (50 m × 0.25 mm inner diameter), and a flame-ionization detector (Agilent Technologies, USA). The analysis was made in the stable detector temperature (250°C) and changing chromatograph column temperature (change from 160°C to 225°C in the rate of 5°C/min). Individual fatty acids and standards were identified based on retention times. Total content of CER, DAG, and TAG was estimated as a sum of identified long-chain fatty acid species, and it was expressed in nanomoles per gram of the muscle weight, while FFA level was expressed in nanomoles per milliliter of the plasma. An exemplary chromatogram has been presented in Figure 1.

### 2.4. Protein Extraction and Western Blot

Routine Western blotting procedures were used to detect protein content as described previously [38–40]. The samples were homogenized in ice-cold RIPA (radioimmunoprecipitation assay) buffer (50 mM Tris-HCl, 150 M NaCl, 1 mM EDTA, 1% NP-40, 0.25% Na-deoxycholate, 1 mM phenylmethylsulfonyl fluoride, 1 *μ*g/ml aprotinin, 1 *μ*g/ml leupeptin, 1 *μ*g/ml pepstatin, 1 mM sodium orthovanadate, and 1 mM sodium fluoride) for 1 min at 4°C. Protein concentration was determined using BCA protein assay kit with bovine serum albumin as a standard. Samples were boiled at 95°C for 10 minutes in a sample buffer containing 2-mercaptoethanol. Protein (40 *μ*g) was subjected to SDS-PAGE and transferred to PVDF membranes, followed by blocking membranes in TTBS buffer (50 mM Tris-HCl, 130 mM NaCl, and 0.05% Tween-20) containing 5% nonfat dry milk for 90 min at room temperature. The membranes were then incubated overnight at 4°C with the corresponding primary antibodies. Antibodies were purchased from Santa Cruz Biotechnology (FAT/CD36, FATP1, FATP4, and GAPDH). Thereafter, the membranes were incubated with anti-rabbit IgG horseradish peroxidase-conjugated secondary antibody (1 : 3000; Santa Cruz Biotechnology, USA). Immunoreactive protein bands were visualized by using an enhanced chemiluminescence substrate (Thermo Scientific, USA) and quantified by densitometry (Bio-Rad, USA). Equal protein concentrations were loaded in each lane as confirmed by Ponceau staining on blot membranes. Protein expression was normalized to GAPDH. Finally, the control was set to 100% and the experimental groups were expressed relatively to the control.

### 2.5. Statistical Analysis

All results are placed in the tables and/or graphs with the mean corresponding to bar height and SEM (standard error of the mean) to whiskers. Statistical differences were tested using ANOVA with a post hoc test (pairwise Student's *t*-test). Alternatively, if their assumptions did not hold, nonparametric Kruskall-Wallis test with the following pairwise Wilcoxon test was applied. The obtained *p* values were adjusted with respect to multiple comparisons (Benjamini-Hochberg correction). The differences with *p* values below 0.05 were considered to be statistically significant.

## 3. Results

### 3.1. Effects of Streptozotocin (STZ), High-Fat Diet (HFD), and Myriocin on Body Weight, Fasting Serum Glucose, Insulin, and FFA Levels (Table 1)

Streptozotocin administration caused a drop (−27%, STZ M− versus Ctrl M−, *p* < 0.05), while the diet regime an increase (+21%, HFD M− versus Ctrl M−, *p* < 0.05) in the animals' body mass. Interestingly, application of myriocin led to a decrement in the rats' body mass in all groups (Table 1, *p* < 0.05).

Both STZ and HFD groups had increased plasma glucose levels (+4 fold and +55%, STZ M− and HFD M− versus Ctrl M−, respectively, *p* < 0.05). Myriocin caused a significant reduction of blood glucose level but only in the case of STZ and HFD rats (−49% and −30%, STZ M+ versus STZ M− and HFD M+ versus HFD M−, respectively, *p* < 0.05). STZ treatment caused a decline, below the level of detection, in the blood insulin level, whereas the HFD significantly increased plasma insulin concentration (+11.5 fold, HFD M− versus Ctrl M−, *p* < 0.05) which was alleviated by myriocin application (−38% HFD M+ versus HFD M−, *p* < 0.05).

Equally, the STZ and HFD increased levels of plasma FFA (+51% and +64%, STZ M− and HFD M− versus Ctrl M−, respectively, *p* < 0.05). Myriocin administration slightly increased plasma FFA level but only in the case of HFD-fed animals (+16%, HFD M+ versus HFD M−, *p* < 0.05).

### 3.2. Effects of Streptozotocin (STZ), High-Fat Diet (HFD), and Myriocin on Diaphragm Expression of Fatty Acid (FAT/CD36, FATP-1, and FATP-4) (Figures 2, 3, and 4)

**Figure 2:**
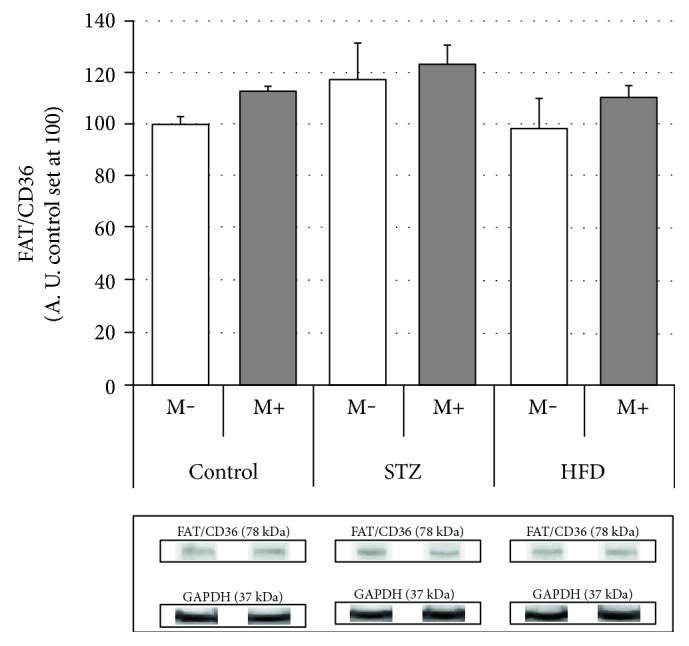
Effects of streptozotocin (STZ), high-fat diet (HFD), and myriocin (M) on diaphragm FAT/CD36 expression (A. U.: arbitrary units, control set at 100, *n* = 6 per group). No statistically significant changes versus control (Ctrl) or within group differences (M+ versus M−) were noticed, *p* < 0.05.

Neither streptozotocin nor high-fat diet had any influence on diaphragm FAT/CD36 expression, since we found no differences between the studied groups. Moreover, myriocin treatment appears to be inert with respect to FAT/CD36 diaphragm content as we observed none within groups differences (Figure 2).

**Figure 3:**
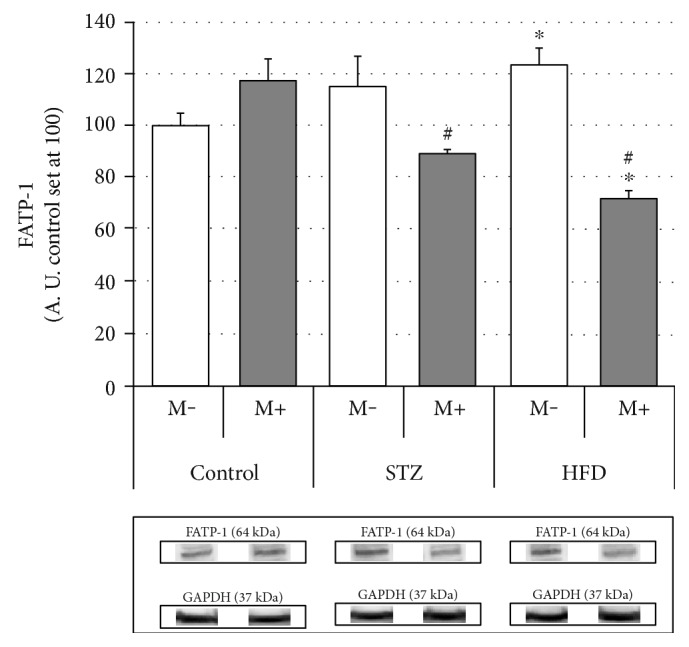
Effects of streptozotocin (STZ), high-fat diet (HFD), and myriocin (M) on diaphragm FATP-1 expression (A. U.: arbitrary units, control set at 100, *n* = 6 per group). ^∗^Difference versus control (Ctrl); ^#^within group difference (M+ versus M−), *p* < 0.05.

As shown in Figure 3, there were no differences between STZ and the controls with respect to the diaphragm FATP-1 expression. In the case of HFD, however, we observed an increase (+23%, HFD M− versus Ctrl M−, *p* < 0.05). Interestingly, myriocin application caused significant decrements in the diaphragm FATP-1 expression, but only in the case of streptozotocin and high-fat diet-fed rats (−22% and −42%, STZ M+ versus STZ M− and HFD M+ versus HFD M−, respectively, *p* < 0.05).

**Figure 4:**
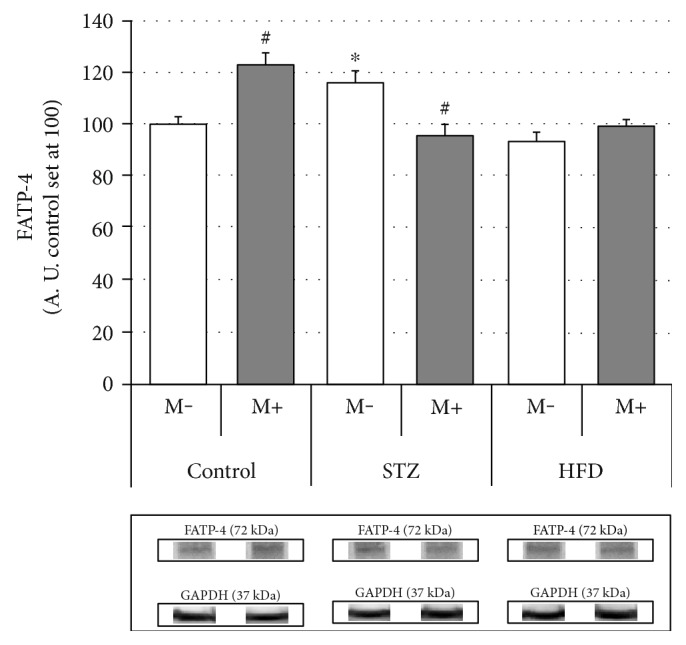
Effects of streptozotocin (STZ), high-fat diet (HFD), and myriocin (M) on diaphragm FATP-4 expression (A. U.: arbitrary units, control set at 100, *n* = 6 per group). ^∗^Difference versus control (Ctrl); ^#^within group difference (M+ versus M−), *p* < 0.05.

We observed an increased expression of FATP-4, in the group treated with streptozotocin (+17%, STZ M− versus Ctrl M−, *p* < 0.05). Surprisingly, application of myriocin contributed to a decrement of the diaphragm FATP-4 content in the STZ group (−18%, STZ M+ versus STZ M−, *p* < 0.05), but caused an increment in the case of control animals (+23%, Ctrl M+ versus Ctrl M−, *p* < 0.05) (Figure 4).

### 3.3. Effects of Streptozotocin (STZ), High-Fat Diet (HFD), and Myriocin on Diaphragm Lipid (TAG, DAG, and CER) Content (Figures 5, 6, and 7, Tables 2, 3, and 4)

**Figure 5:**
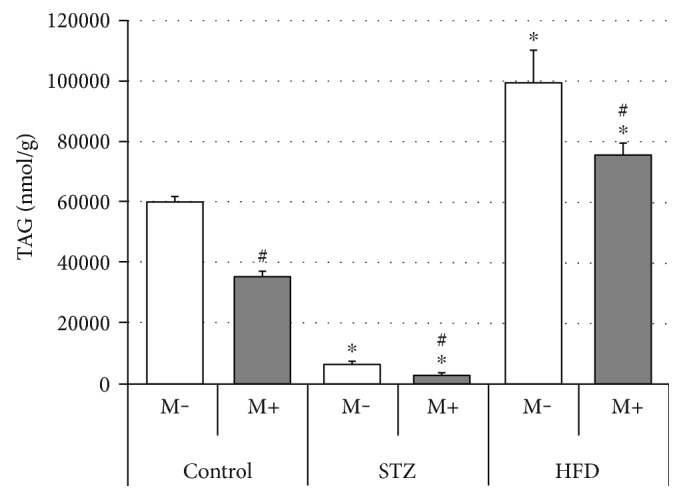
Effects of streptozotocin (STZ), high-fat diet (HFD), and myriocin (M) on diaphragm triacylglycerol (TAG) content (mean ± SEM, *n* = 6 per group). ^∗^Difference versus control (Ctrl); ^#^within group difference (M+ versus M−), *p* < 0.05.

As shown in Figure 5, the rats from the STZ group had decreased, whereas their counterparts from the HFD group increased, diaphragm triacylglycerol content (−99% and +66%, STZ M− and HFD M− versus Ctrl M−, respectively, *p* < 0.05). Moreover, myriocin administration significantly decreased TAG content in all of the groups (−41%, −54%, and −24%, Ctrl M+ versus Ctrl M−, STZ M+ versus STZ M−, and HFD M+ versus HFD M−, respectively, *p* < 0.05). Additionally, in comparison with the control group (Ctrl M−), STZ treatment led to a change in the content of (1) saturated (SAT), that is, myristic (−95%), palmitic (−92%), and stearic (−83%), and (2) unsaturated (UNSAT), that is, palmitooleic (−99.9%), oleic (−92%), linoleic (−70%), linolenic (−80%), arachidonic (−38%), and docosahexaenoic (+175%), fatty acids. Similarly, HFD treatment also caused significant alterations with respect to (1) SAT, that is, stearic (+187%), arachidic (+115%), behenic (+89%), and lignoceric (+73%), and (2) UNSAT, that is, palmitooleic (−66%), oleic (+112%), linoleic (+243%), linolenic (+148%), arachidonic (+70%), eicosapentaenoic (+134%), nervonic (+129%), and docosahexaenoic (+77%), fatty acid composition (Table 3).

**Figure 6:**
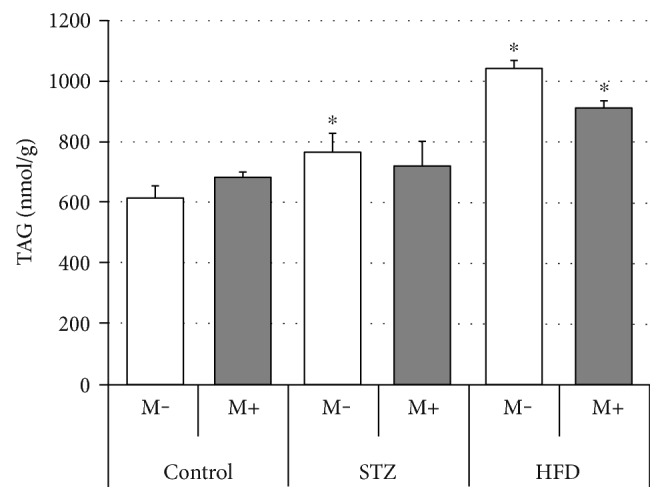
Effects of streptozotocin (STZ), high-fat diet (HFD), and myriocin (M) on diaphragm diacylglycerol (DAG) content (mean ± SEM, *n* = 6 per group). ^∗^Difference versus control (Ctrl); no statistically significant within group differences (M+ versus M−) were noticed.

With respect to the diaphragm diacylglycerol concentration (Figure 6), we have observed an increased content of the fraction in the case of both STZ and HFD groups (+25% and +70%, STZ M− and HFD M− versus Ctrl M−, respectively, *p* < 0.05). Moreover, myriocin treatment exerted virtually no effect on diaphragm DAG accumulation (Figure 6). Additionally, in comparison with the control group (Ctrl M−), STZ treatment led to a change in the content of (1) saturated (SAT), that is, myristic (+51%), stearic (+40%), behenic (+80%), and lignoceric (+65%), and (2) unsaturated (UNSAT), that is, palmitooleic (−74%), linoleic (+54%), arachidonic (+78%), and docosahexaenoic (+78%), fatty acids. Similarly, HFD treatment also caused significant alterations with respect to (1) SAT, that is, myristic (+46%), palmitic (+38%), and behenic (+96%), and (2) UNSAT, that is, oleic (+180%), linoleic (+192%), linolenic (+107%), arachidonic (+34%), nervonic (+132%), and docosahexaenoic (+67%), fatty acid composition (Table 2).

**Figure 7:**
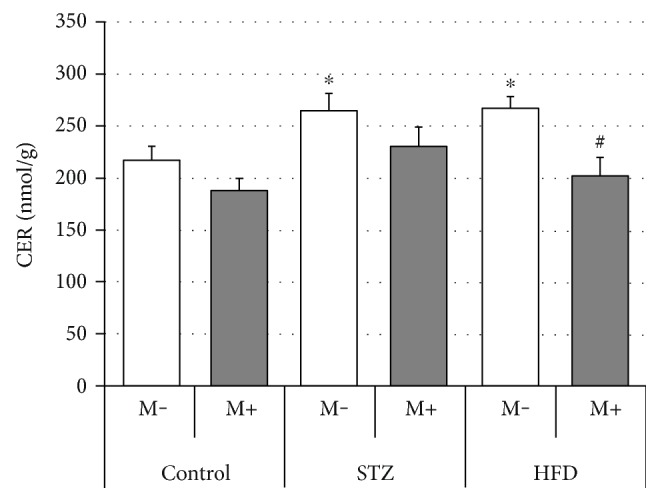
Effects of streptozotocin (STZ), high-fat diet (HFD), and myriocin (M) on diaphragm ceramide (CER) content (mean ± SEM, *n* = 6 per group). ^∗^Difference versus control (Ctrl); ^#^within group difference (M+ versus M−), *p* < 0.05.

We observed significant increases in the diaphragm ceramide levels in the STZ and HFD group in comparison with the control animals (+22% and +23%, STZ M− and HFD M− versus Ctrl M−, respectively, *p* < 0.05, Figure 7). Moreover, myriocin administration lowered ceramide level in all groups (−14%, Ctrl M+ versus Ctrl M−, −13% STZ M+ versus STZ M−, and −24% HFD M+ versus HFD M−), although only in this last case it reached statistical significance level (*p* < 0.05). Additionally, in comparison with the control group (Ctrl M−), STZ treatment led to a change in the content of (1) saturated (SAT), that is, myristic (+44%), palmitic (+31%), arachidic (+100%), behenic (+110%), and lignoceric (+112%), and (2) unsaturated (UNSAT), that is, nervonic (+21%), fatty acids. Similarly, HFD treatment also caused significant alterations with respect to SAT, that is, stearic (+48%), arachidic (+84%), behenic (+69%), and lignoceric (+112%), but not unsaturated (UNSAT) fatty acid composition (Table 4).

## 4. Discussion

In the first step of our procedure, we confirmed the effectiveness of our models by the assessment of fasting blood glucose and insulin levels as well as evaluation of HOMA-IR (Table 1). According to our expectations, we found increased glucose plasma levels in the rats from STZ- and HFD-treated groups accompanied by a dramatically decreased (STZ) and significantly increased (HFD) insulin levels. Moreover, the results were similar to the ones established in the literature by us and other authors and confirm the destruction of the pancreatic *β*-islet cells after STZ injection [12], whereas HFD feeding led to the subsequent development of insulin resistance development [27, 41]. Additionally, we decided upon application of myriocin, a robust, selective inhibitor of ceramide de novo synthesis and, therefore, a potent agent ameliorating insulin resistance [12, 29, 42]. Myriocin treatment noticeably improved glucose metabolism via reduction of hyperglycemia together with improvements in insulin levels and HOMA index (Table 1). A possible explanation of this phenomenon is an improvement of the liver and/or skeletal muscle metabolism, since myriocin acts by alleviating steatosis in both tissues, causing greater glucose uptake and glycogen synthesis [13, 29, 43], and thus contributing to a decreased glucose production [44] and better glucose clearance from the blood. Collectively, the basic characteristics of the groups presented in Table 1 confirm the effectiveness and appropriateness of our experimental model, that is, successful induction of type 1 diabetes (STZ) and overall insulin resistance (HFD).

We observed an increased ceramide level in the diaphragms of rats that underwent STZ or HFD treatment, which agrees with the previous findings for oxidative/oxidative-glycolytic skeletal muscles like soleus and red gastrocnemius [12, 30] and is a strong indicator of a muscle insulin resistance [5, 29]. Interestingly, not only did we find an increased total ceramide content but also its fatty acid moieties, that is, stearic (C18 : 0) and palmitic (C16:0) acids, in both STZ and HFD groups (Table 4). This seems to be of vital importance, since previous studies demonstrated that ceramide saturated fatty acids in general, and long chain saturated fatty acids (≥16 carbon atoms) in particular, correlate well with the development of muscular insulin resistance [45–47]. Chavez and Summers, for instance, demonstrated that the accumulation of palmitic and stearic acids is a potent inhibitor of Akt/PKB activation and therefore whole insulin signaling pathway [47]. Interestingly, most of the fatty acid species levels were increased in our study in both STZ and HFD groups (Table 4). Moreover, also other findings from this study, that is, increased diacylglycerol concentration (Figure 6) in both experimental groups as well as enhanced TAG accumulation in the HFD-fed animals (Figure 5) appears to confirm that last notion. Furthermore, we observed an increased content of saturated fatty acids constituting diacylglycerol lipid fraction (Table 2). This finding seems to be consistent with the prior studies on skeletal muscle cells conducted by Montell et al. [48] and Chavez and Summers [47]. Moreover, it fits in the previously postulated role of the increased accumulation of saturated fatty acids in the insulin resistance development. Montell and coworkers, for instance, showed an increased incorporation of unsaturated fatty acids into TAG fraction, whereas their saturated counterparts were mostly directed towards DAG synthesis [48]. Likewise, Chavez and Summers demonstrated an increased incorporation of myristic (C 14 : 0), palmitic (C16:0), stearic (C18 : 0), arachidic (C20:4), and lignoceric (C24 : 0) acids into diacylglycerol fraction which was associated with the development of insulin resistance [47]. Interestingly, most of the abovementioned DAG's fatty acid species were increased in the diaphragms of STZ and/or HFD rats (Table 2).

Surprisingly, we observed a severely decreased diaphragm TAG concentration in the STZ group (−99% STZ M− versus Ctrl M−, *p* < 0.05, Figure 5). Moreover, the observed change was caused by a drop in the amount of most of the fatty acid species composing TAG (Table 3). This is rather unexpected, given that previous studies on skeletal muscles indicate rather increased TAG intramuscular content in response to type 1 diabetes [12, 14, 15]. Furthermore, due to the scarcity of data regarding lipid metabolism in the diaphragm, it is rather hard to satisfactorily explain this finding. Nevertheless, we found research [49] in which enzymatic activities of several proteins involved in lipid oxidation showed a significantly increased activity (+60% of 3-hydroxyacyl-CoA dehydrogenase) in the diaphragms of type 1 diabetic Sprague-Dawley rats. This finding seems to be in accordance with the report on gene expression profile in the diaphragms of Wistar rats with type 1 diabetes published by van Lunteren and Moyer [25]. The abovementioned study revealed a decreased expression (−2.7 fold change, *p* < 0.05) of acyl-CoA synthase (Acsl6, lipid synthetizing enzyme) and an increased expression (+4.2 fold) of mitochondrial acyl-CoA thioesterase (Mte1, auxiliary enzyme in lipid oxidation) [25]. Taken altogether, these data indicate a probable and quite significant increase in the FA *β*-oxidation (HADH, Mte1) in connection with FA decreased production (Ascl6) in the diaphragms of type 1 diabetic animals. The above, in turn, could well translate into lower diaphragm TAG content, since intramuscular triacylglycerols are one of the main energy sources for skeletal muscles, in the rats with type 1 diabetes observed in our study (Figure 5).

Closer inspection of the results obtained in this study (Figures 2–7) seems to indicate slightly different response pattern of the diaphragm in the case of type 1 diabetic (STZ) as compared with the prediabetic rats (HFD). Both pathological conditions are characterized by greater concentrations of bioactive lipids (CER and DAG; Figures 6 and 7) and larger FA influx [increased expression of FATP-1 in the HFD group (Figure 3) and FATP-4 in the STZ group (Figure 4)]. The diaphragms of the HFD-fed animals, however, have these changes more pronounced, that is, greater CER content than in the STZ rodents (Figure 7); moreover, they seem to reap greater benefits from myriocin treatment (significant reductions of DAG and CER content, Figures 6 and 7). Altogether, it could signify that (a) both treatments (STZ and HFD) may lead to the development of diaphragm insulin resistance and (b) the degree of the insulin resistance is greater in the case of HFD. The existence of such a phenomenon in the diaphragm is in agreement with previously conducted studies [12, 14, 30], since similar pattern of changes, with respect to IR lipid biomarkers, was noticed for both (pre- and diabetic conditions) in the soleus and red gastrocnemius muscle [12, 30]. Moreover, the existence of insulin resistance in human type 1 diabetes was confirmed by Perseghin and coworkers [14]. The authors observed a reduced insulin-stimulated glucose clearance rate (MCR: 5.1 ± 0.6 ml·kg^−1^·min^−1^) in T1DM patients. This classified them between the healthy subjects (MCR: 8.5 ± 0.5 ml·kg^−1^·min^−1^) and the insulin-resistant individuals with type 2 diabetes mellitus (3.2 ± 0.8 ml·kg^−1^·min^−1^) [14]. Moreover, the decreased muscular insulin sensitivity in the type 1 diabetic patients was connected with the increased soleus IMCL content [14]. Furthermore, the conducted analysis revealed the existence of a positive correlation between the soleus IMCL level and blood glycated hemoglobin HbA1C [used as an indicator of glycemia level in diabetes] (*r* ≈ 0.75, *r*^2^ = 0.57, *p* < 0.001) [14]. This seems to fit as an explanation of the results obtained in our study although we must bear in mind that in the case of insulin-dependent diabetes, lipid accumulation and consequent skeletal muscle insulin resistance are considered as a secondary phenomenons [14, 17].

Additionally, in agreement with our previous research for skeletal muscles [29, 30], we found that myriocin administration tends to reduce the diaphragm accumulation of ceramide, diacylglycerol, and triacylglycerol, especially in the case of HFD rats (Figures 5–7). Noticeably, in the diaphragm, both magnitude of changes and their reduction after myriocin application seem to be smaller than in other skeletal muscles, for example, soleus [12, 30]. With respect to ceramide, we observed only modest +22% (Figure 7) increment of its level (in comparison with +50–75% changes observed in other studies in muscle tissue [12, 30]). Moreover, application of myriocin also produced only a moderate (14%–24%, Figure 7) reduction (in comparison with 75% downfall reported elsewhere [12, 30]). Perhaps, the explanation of this finding (smaller degree of lipid pool changes) lies in the nature of the diaphragm, since it is characterized by an uninterrupted cyclic contraction pattern [18]. The pattern makes it somewhat similar, at least with respect to contraction rhythmicity, to the heart and imposes high tissue lipid turnover. In line with that notion, a recent study of Harasim et al. [50] showed that the hearts of high-fat-fed rats presented only a modest response to a dietary regime with respect to their ceramide content (+31%, HFD week 5 versus HFD week 0, *p* < 0.05) and no statistically significant changes with respect to diacylglycerol concentration [50]. Interestingly, some authors found a compensatory increase in the diaphragm activity which is presented in obese patients [26]. This is also in line with the proposed increased lipid turnover explanation, since it was demonstrated that skeletal muscles of trained Wistar rats present no or slight changes with respect to sphingolipid (e.g., ceramide) concentration [51].

In conclusion, there are several novel findings reported in the current study. We found that both streptozotocin administration and high-fat diet regime may induce insulin resistance in the diaphragm muscle of Wistar rats. The extent of IR, however, seems to be relatively modest in the respiratory muscle of type 1 diabetic rats. Moreover, and quite surprisingly, the rats from STZ group had a significantly lower TAG content (−99% STZ M− versus Ctrl M−).

## Figures and Tables

**Table 1:** Effects of high-fat diet (HFD), streptozotocin (STZ), and myriocin (M) on body weight, fasting serum glucose, insulin, and FFA levels in Wistar rats (mean ± SEM, *n* = 6 per group). ^∗^Difference versus Ctrl (*p* < 0.05); ^#^within group difference (M− versus M+) (*p* < 0.05). ND: not detected.

	Ctrl		STZ		HFD	
	M−	M+	M−	M+	M−	M+
Initial body mass (g)	86.1 ± 4.65	89.8 ± 4.83	86.6 ± 4.42	87.1 ± 3.65	88.3 ± 4.40	83.8 ± 5.83
Final body mass (g)	312.1 ± 10.25	244.8 ± 9.63^#^	228.2 ± 7.47^∗^	177.9 ± 5.04^∗^^#^	377.5 ± 13.35^∗^	283.2 ± 7.21^∗^^#^
Plasma glucose (mg/dl)	104.8 ± 3.65	99.6 ± 4.03	526.8 ± 14.49^∗^	323.3 ± 11.2^∗^^#^	162.6 ± 8.36^∗^	113.1 ± 7.61^#^
Plasma insulin (*μ*U/ml)	4.4 ± 0.22	4.8 ± 0.30	ND	ND	55 ± 3.16^∗^	38.1 ± 2.46^∗^^#^
HOMA-IR	1.2 ± 0.09	1.2 ± 0.11	—	—	22.3 ± 2.20^∗^	9.5 ± 0.31^∗^^#^
Plasma FFA (*μ*mol/l)	98.6 ± 3.54	108.4 ± 5.04	148.8 ± 10.32^∗^	148.6 ± 10.74^∗^	162.0 ± 7.12^∗^	187.4 ± 6.78^∗^^#^

**Table 2:** Effects of high-fat diet (HFD), streptozotocin (STZ), and myriocin (M) on diaphragm diacylglycerol content (mean ± SEM, *n* = 6 per group). ^∗^Difference versus Ctrl (*p* < 0.05); ^#^within group difference (M+ versus M−) (*p* < 0.05). ND: not detected.

	DAG fatty acid composition (nmol/g of wet tissue)					
	Ctrl		STZ		HFD	
	M−	M+	M−	M+	M−	M+
Myristic acid (14 : 0)	41.16 ± 3.421	33.47 ± 0.945	65.32 ± 5.367^∗^	64.19 ± 8.595	59.98 ± 2.605^∗^	56.98 ± 3.174^∗^
Palmitic acid (16 : 0)	234.9 ± 17.416	226.84 ± 7.371	276.12 ± 21.028	245.71 ± 27.51	324.53 ± 17.959^∗^	280.31 ± 7.954
Palmitooleic acid (16 : 1)	28.81 ± 5.212	15.75 ± 1.367	7.58 ± 0.399^∗^	7.52 ± 0.797^∗^	20.6 ± 1.744	17.22 ± 1.493
Stearic acid (18 : 0)	135.01 ± 13.544	142.8 ± 8.175	189.69 ± 31.681^∗^	170.03 ± 21.512	170.38 ± 4.55	164.04 ± 20.206
Oleic acid (18:1n9c)	80.08 ± 7.586	99.81 ± 3.947	82.19 ± 3.315	82.63 ± 6.384	224.42 ± 11.243^∗^	179.07 ± 15.183^∗^^#^
Linoleic acid (18:2n6c)	67.48 ± 3.321	128.42 ± 5.649^#^	103.91 ± 3.415^∗^	107.32 ± 9.995^∗^	197.68 ± 5.589^∗^	174.29 ± 12.654^∗^
Arachidic acid (20 : 0)	5.4 ± 1.08	4.51 ± 0.267	7.06 ± 0.69	6.81 ± 0.945	7.17 ± 0.836	5.59 ± 0.436
Linolenic acid (C18:9n3)	5.07 ± 0.731	8.2 ± 0.32^#^	6.3 ± 0.301	6.74 ± 0.481^∗^	10.48 ± 0.33^∗^	8.6 ± 0.446^∗^^#^
Behenic acid (22 : 0)	1.67 ± 0.157	1.71 ± 0.108	3.02 ± 0.211^∗^	3.02 ± 0.436^∗^	3.27 ± 0.659^∗^	2.36 ± 0.272
Arachidonic acid (20:4n6)	7.37 ± 0.925	15.43 ± 0.863^#^	13.12 ± 0.868^∗^	12.09 ± 0.568^∗^	9.86 ± 0.447^∗^	11.55 ± 0.663^∗^
Lignoceric acid (24 : 0)	4.79 ± 0.566	4.6 ± 0.481	7.93 ± 0.485^∗^	8.6 ± 0.39^∗^	8.85 ± 2.63	5.86 ± 0.589
Eicosapentaenoic acid (20:5n3)	ND	ND	ND	ND	ND	ND
Nervonic acid (24 : 1)	0.61 ± 0.157	1.64 ± 0.091^#^	1.05 ± 0.091	1.25 ± 0.288	1.42 ± 0.32^∗^	1.46 ± 0.153^∗^
Docosahexaenoic acid (22:6n3)	3.83 ± 0.491	2.46 ± 0.246	5.98 ± 0.39^∗^	6.85 ± 0.8^∗^	6.35 ± 0.431^∗^	6.33 ± 0.56^∗^
SAT	422.92 ± 33.997	413.94 ± 12.605	549.14 ± 56.728	498.37 ± 58.127	574.19 ± 15.28^∗^	515.13 ± 25.606
UNSAT	193.26 ± 14.719	271.72 ± 9.722^#^	220.11 ± 6.939	224.4 ± 18.456	470.82 ± 15.253^∗^	398.53 ± 29.846^∗^^#^
Total	616.18 ± 37.719	685.66 ± 15.206	769.26 ± 57.27^∗^	722.77 ± 76.397	1045.01 ± 22.682^∗^	913.66 ± 22.965^∗^

**Table 3:** Effects of high-fat diet (HFD), streptozotocin (STZ), and myriocin (M) on diaphragm triacylglycerol content (mean ± SEM, *n* = 6 per group). ^∗^Difference versus Ctrl (*p* < 0.05); ^#^within group difference (M+ versus M−) (*p* < 0.05). ND: not detected.

	TAG fatty acid composition (nmol/g of wet tissue)					
	Ctrl		STZ		HFD	
	M−	M±	M−	M±	M−	M±
Myristic acid (14 : 0)	1730.1 ± 82.76	838.9 ± 48.91^#^	83.3 ± 5.3^∗^	45.4 ± 3.27^∗^^#^	1607.6 ± 159.74	1148.5 ± 54.63^∗^^#^
Palmitic acid (16 : 0)	21948.8 ± 675.9	10,583 ± 605.94^#^	1850.9 ± 188.69^∗^	802.6 ± 104.53^∗^^#^	23838.9 ± 3244	17682.7 ± 863^∗^
Palmitooleic acid (16 : 1)	7945.4 ± 395.71	1992.8 ± 140.51^#^	74.7 ± 3^∗^	40 ± 7.66^∗^^#^	2731.7 ± 423.93^∗^	2427 ± 356.94^∗^
Stearic acid (18 : 0)	2394.1 ± 101.64	1484.9 ± 46.57^#^	400.8 ± 22.51^∗^	239.9 ± 23.26^∗^^#^	6880.6 ± 635.14^∗^	4798.4 ± 163.82^∗^^#^
Oleic acid (18:1n9c)	17958.7 ± 595.07	9165.1 ± 266.3^#^	1455.8 ± 130.44^∗^	638.2 ± 72.06^∗^^#^	38246.9 ± 3548.67^∗^	28022.8 ± 1580.96^∗^^#^
Linoleic acid (18:2n6c)	7011 ± 472.89	10364.3 ± 531.09^#^	2092.5 ± 161.89^∗^	865.3 ± 125.93^∗^^#^	24048.4 ± 2718.94^∗^	19,753 ± 1685.25^∗^
Arachidic acid (20 : 0)	38.2 ± 3.49	28.8 ± 1.82	31.5 ± 1.19	17.3 ± 1.51^∗^^#^	82.3 ± 4.56^∗^	86 ± 6.2^∗^
Linolenic acid (C18:9n3)	580.7 ± 40.41	674.5 ± 53.83	118.3 ± 7.24^∗^	57.6 ± 9.51^∗^^#^	1440.7 ± 194.71^∗^	1057.3 ± 118.49^∗^
Behenic acid (22 : 0)	25.1 ± 4.29	15.4 ± 0.86	30.8 ± 1.92	17.2 ± 2.06^#^	47.4 ± 3.67^∗^	53.2 ± 6.21^∗^
Arachidonic acid (20:4n6)	177.3 ± 15.77	233.2 ± 22.01^#^	110.6 ± 16.02^∗^	71 ± 12.28^∗^	300.9 ± 12.57^∗^	211.9 ± 13.32^#^
Lignoceric acid (24 : 0)	39.6 ± 1.61	20.8 ± 1.35^#^	42.7 ± 15.3	19.7 ± 0.67^∗^^#^	68.4 ± 2.94^∗^	74.6 ± 9.86
Eicosapentaenoic acid (20:5n3)	16.0 ± 2.74	10.1 ± 0.85	9.7 ± 1.39	4.6 ± 0.79^∗^^#^	37.4 ± 1.11^∗^	35.4 ± 5.69^∗^
Nervonic acid (24 : 1)	17.4 ± 1.82	9.6 ± 0.97^#^	15.3 ± 0.65	8.1 ± 0.65^∗^^#^	39.9 ± 3.59^∗^	46.3 ± 5.96^∗^
Docosahexaenoic acid (22:6n3)	129.2 ± 14.46	174.2 ± 12.55	355.3 ± 28.67^∗^	228.2 ± 41.62^∗^^#^	272.3 ± 19.49^∗^	203.8 ± 19.63
SAT	26175.8 ± 787.38	12971.7 ± 620.66^#^	2439.9 ± 201.06^∗^	1142.1 ± 128.61^∗^^#^	32525.1 ± 4017.93	23843.3 ± 1055.22
UNSAT	33835.8 ± 1081.05	22623.7 ± 659.81^#^	4232.2 ± 337.45^∗^	1912.9 ± 253.3^∗^^#^	67118.3 ± 6608.54^∗^	51757.6 ± 2808.14^∗^^#^
Total	60011.6 ± 1817.28	35595.4 ± 1203.21^#^	6672.1 ± 535.69^∗^	3055 ± 379.25^∗^^#^	99643.4 ± 10495.24^∗^	75600.9 ± 3772.94^∗^^#^

**Table 4:** Effects of high-fat diet (HFD), streptozotocin (STZ), and myriocin (M) on diaphragm ceramide content (mean ± SEM, *n* = 6 per group). ^∗^Difference versus Ctrl (*p* < 0.05); ^#^within group difference (M+ versus M−) (*p* < 0.05). ND: not detected.

	CER fatty acid composition (nmol/g of wet tissue)					
	Ctrl		STZ		HFD	
	M−	M+	M−	M+	M−	M+
Myristic acid (14 : 0)	8.4 ± 0.85	6 ± 0.45^#^	12.1 ± 0.6^∗^	9 ± 0.71^#^	8.7 ± 0.48	7.3 ± 0.84
Palmitic acid (16 : 0)	53.2 ± 3.24	43.3 ± 2.77	69.8 ± 6.39^∗^	51.6 ± 4.04^#^	59.4 ± 2.16	47.3 ± 4.35
Palmitooleic acid (16 : 1)	3.6 ± 0.28	2.6 ± 0.21	4 ± 0.26	4 ± 0.37	3.8 ± 0.21	3.3 ± 0.26
Stearic acid (18:0)	59.4 ± 3.41	63 ± 5.41	72.6 ± 7.8	57.9 ± 5.38	88.1 ± 4.27^∗^	55.7 ± 4.14^#^
Oleic acid (18:1n9c)	28 ± 1.85	19.9 ± 0.87^#^	26.8 ± 0.86	29.3 ± 1.57	26.7 ± 1.57	26.1 ± 1.94
Linoleic acid (18:2n6c)	34.2 ± 2.2	22.8 ± 1.52^#^	34.2 ± 1.26	40.6 ± 3.09	35.9 ± 3.11	31.7 ± 2.12
Arachidic acid (20 : 0)	3.1 ± 0.19	3.9 ± 0.32	6.2 ± 0.08^∗^	4.8 ± 0.47^∗^^#^	5.7 ± 0.24^∗^	3.5 ± 0.51^#^
Linolenic acid (C18:9n3)	1.5 ± 0.09	1 ± 0.11^#^	1.6 ± 0.07	2 ± 0.21^∗^	1.6 ± 0.09	1.6 ± 0.1
Behenic acid (22 : 0)	3.9 ± 0.13	4.3 ± 0.48	8.2 ± 0.16^∗^	5.8 ± 0.53^∗^^#^	6.6 ± 0.44^∗^	4.4 ± 0.84
Arachidonic acid (20:4n6)	2.6 ± 0.74	1.4 ± 0.25	2.6 ± 0.38	3.8 ± 0.63	4.6 ± 1.08	4.6 ± 0.92
Lignoceric acid (24 : 0)	7.3 ± 0.28	12.7 ± 0.76^#^	15.5 ± 0.33^∗^	10.6 ± 1.13^∗^^#^	15.5 ± 0.52^∗^	8.1 ± 0.79^#^
Eicosapentaenoic acid (20:5n3)	2.9 ± 0.16	ND	ND	ND	ND	ND
Nervonic acid (24 : 1)	5.7 ± 0.23	4.8 ± 0.45	6.9 ± 0.11^∗^	6.2 ± 0.34	6.8 ± 0.39	5.8 ± 0.61
Docosahexaenoic acid (22:6n3)	3.9 ± 0.34	2.6 ± 0.2	4.4 ± 0.31	5.1 ± 0.56	3.9 ± 0.48	3.2 ± 0.45
SAT	135.2 ± 6.97	133.2 ± 8.29	184.6 ± 13.89^∗^	139.8 ± 11.59^#^	184.1 ± 6.13^∗^	126.2 ± 10.81^#^
UNSAT	82.5 ± 5.17	55.1 ± 3.28^#^	80.5 ± 2.26	91 ± 6.25	83.3 ± 6.26	76.4 ± 5.56
Total	217.8 ± 11.75	188.3 ± 10.06	265.1 ± 15.28^∗^	230.7 ± 16.87	267.4 ± 9.71^∗^	202.6 ± 16.11^#^
